# Estimates of lithium mass yields from produced water sourced from the Devonian-aged Marcellus Shale

**DOI:** 10.1038/s41598-024-58887-x

**Published:** 2024-04-16

**Authors:** Justin Mackey, Daniel J. Bain, Greg Lackey, James Gardiner, Djuna Gulliver, Barbara Kutchko

**Affiliations:** 1 National Energy Technology Laboratory, Pittsburgh, PA 15236 USA; 2 NETL Support Contractor, Pittsburgh, PA 15236 USA; 3 University of Pittsburgh, Pittsburgh, PA 15260 USA

**Keywords:** Environmental sciences, Energy science and technology

## Abstract

Decarbonatization initiatives have rapidly increased the demand for lithium. This study uses public waste compliance reports and Monte Carlo approaches to estimate total lithium mass yields from produced water (PW) sourced from the Marcellus Shale in Pennsylvania (PA). Statewide, Marcellus Shale PW has substantial extractable lithium, however, concentrations, production volumes and extraction efficiencies vary between the northeast and southwest operating zones. Annual estimates suggest statewide lithium mass yields of approximately 1160 (95% CI 1140–1180) metric tons (mt) per year. Production decline curve analysis on PW volumes reveal cumulative volumetric disparities between the northeast (median = 2.89 X 10^7^ L/10-year) and southwest (median = 5.56 × 10^7^ L/10-year) regions of the state, influencing lithium yield estimates of individual wells in southwest [2.90 (95% CI 2.80–2.99) mt/10-year] and northeast [1.96 (CI 1.86–2.07) mt/10-year] PA. Moreover, Mg/Li mass ratios vary regionally, where NE PA are low Mg/Li fluids, having a median Mg/Li mass ratio of 5.39 (IQR, 2.66–7.26) and SW PA PW is higher with a median Mg/Li mass ratio of 17.8 (IQR, 14.3–20.7). These estimates indicate substantial lithium yields from Marcellus PW, though regional variability in chemistry and production may impact recovery efficiencies.

## Introduction

Lithium (Li) is a major battery component in electric vehicles (EV) and is part of a broader group of critical elements (minerals) with existing supply chain concerns. Moreover, Li is considered essential to the US economy due to domestic consumption in energy, manufacturing and defense. The Infrastructure Investment and Jobs Act^1^, commonly referred to as the Bipartisan Infrastructure Law, requires the raw materials used in EV battery components to be sourced domestically by 2030. As such, Li demand scenarios from net-zero and decarbonization initiatives could drive global demand of the critical metal up 400%^2^. These factors necessitate alternative domestic sources of Li to reliably enable the energy transition. Recent work has shown the aqueous fluid that is co-produced with hydrocarbons during oil and gas operations, referred to as produced water (PW), has significant potential as an alternative source of Li^3,4^. Specifically, evidence suggests produced waters from Paleozoic stratigraphy of the Appalachian region have economically viable Li concentrations^5–7^.

**Figure 1:**
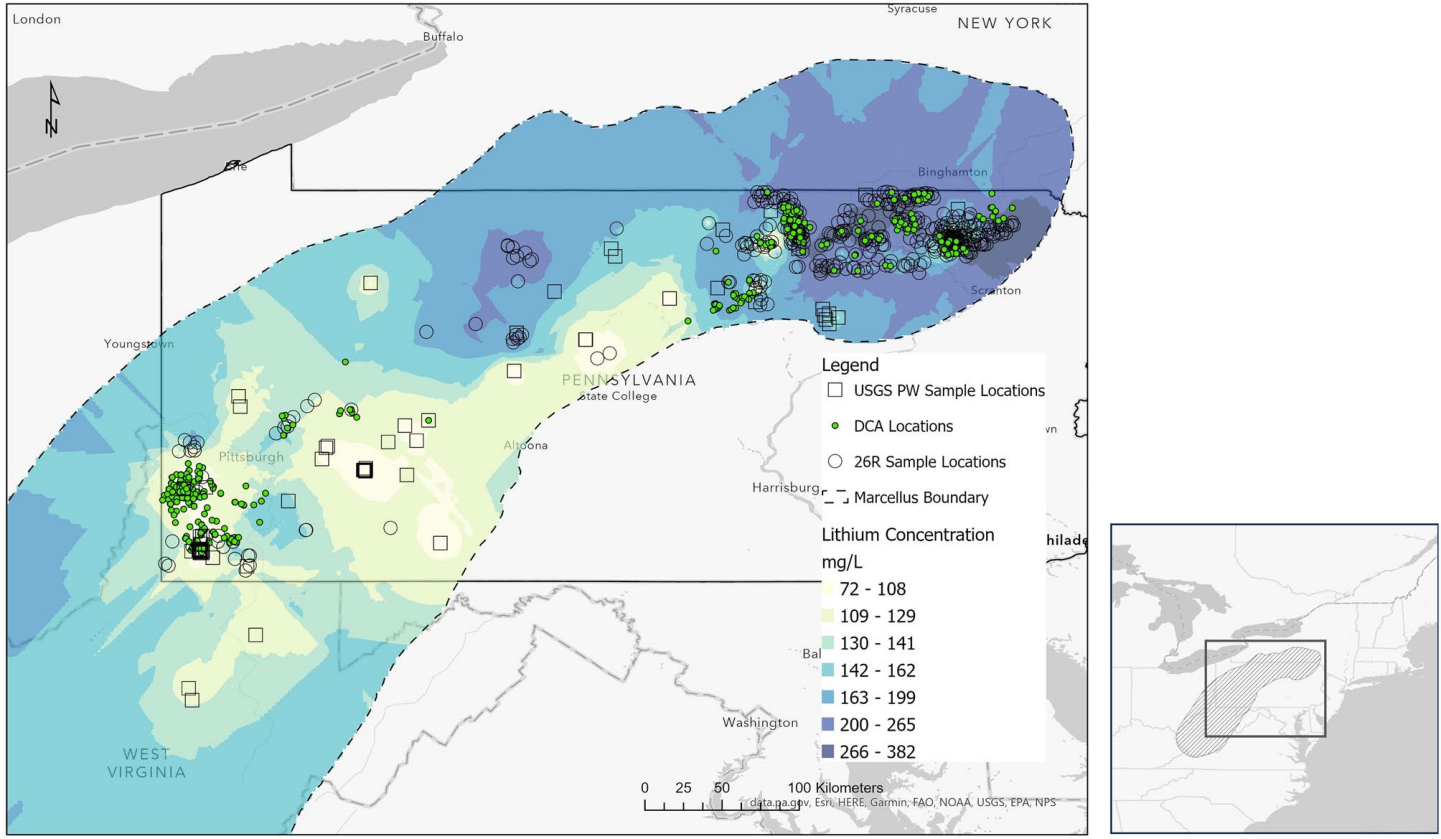
Map of study area showing the Marcellus shale extent, well locations using in decline curve analysis (DCA), PW samples used in this study, and previous USGS sample locations. Lithium (Li) concentration data was calculated using new data reported in this manuscript and existing data from the USGS National Produced Waters Database^13^. This map was generated with ArcGIS Pro 3.1.4 software, ESRI, https://www.esri.com/en-us/arcgis/products/arcgis-pro/overview. Sources of data cited on this map include: “data.pa.gov, ESRI, HERE, Garmin, FAO, NOAA, USGS, EPA and NPS.”

A promising domestic source of Li is PW from Marcellus Formation, a late Paleozoic (Middle-Devonian) aged unconventional natural gas field that underlies significant portions of central Appalachia (Fig. 1). Unconventional formations, such as the Marcellus, require substantial amounts of water to hydraulically fracture the formation to produce hydrocarbons.

Moreover, the drilling boom and subsequent active wells have culminated in large volumes of PW being generated with limited options for beneficial reuse^8,9^. Currently, ~ 95% of the PW co-produced with natural gas from the Marcellus is recycled in ongoing fracking operations^8^, however this fluid is hypersaline, with total dissolved solids (TDS) concentrations exceeding 100,000 mg/L^10,11^ and requires some treatment prior to reinjection^12^. Significantly, this fluid is enriched in Li relative to other formations of comparable TDS^13,14^. Marcellus Shale was deposited contemporaneous to Middle Devonian volcanism and contains interlayered beds of volcanic ash that, through diagenesis, partitioned Li from the volcanic ash into formation pore fluids making it a suitable target for Li extraction^14–17^. Reservoir properties, infrastructure and operational footprints have concentrated a higher density of wells into two regional natural gas production hot spots, one in the northeast (NE PA) and another in the southwest (SW PA) of Pennsylvania^18^ (Fig. 1). The Marcellus Shale varies compositionally and stratigraphically between these two regions, influencing the chemical complexity of its produced waters^11,19,20^. Thus, it is expected the Li extraction potential will also vary between these regions.

To date, comparative analysis of the Li resource potential of Paleozoic brines from global and domestic perspectives have highlighted the prospect of Li extraction from these waters, however these analyses do not consider specific, intra-basin influences on Li yields^3,4,14^. For example, the rate and quantity of PW generated by a given well can vary widely and these spatial variations introduce substantial uncertainty into basin scale Li mass-yield estimates^21^. Likewise, compositional variance in produced water chemistry can impact the method of Li recovery in Li extraction operations, where higher Mg^2+^/Li^+^ mass ratios decrease absorbent and precipitation efficiencies during water treatment for Li removal^22^. Lastly, the volume of water produced from an unconventional well generally declines with time and will impact the ultimate recovery of Li from that location^23,24^. Compounded, these factors can influence the cost, treatment method and overall recovery yield of lithium from produced water.

This study uses chemical and production compliance data reported to the Pennsylvania Department of Environmental Protection (PA DEP) to predict Li mass yields from the Middle-Devonian Marcellus Shale PW^25,26^. These compliance data are used to incorporate intra-regional variations of PW composition, production decline rates and volumes produced in estimating Li mass yields in Pennsylvania. Furthermore, we employ empirical decline curve analysis to identify spatial trends in the estimated ultimate recovery of lithium from individual wells. The estimates presented here were derived using Monte Carlo methods to quantify and reduce the uncertainty of our results^27^.

## Results

**Figure 2:**
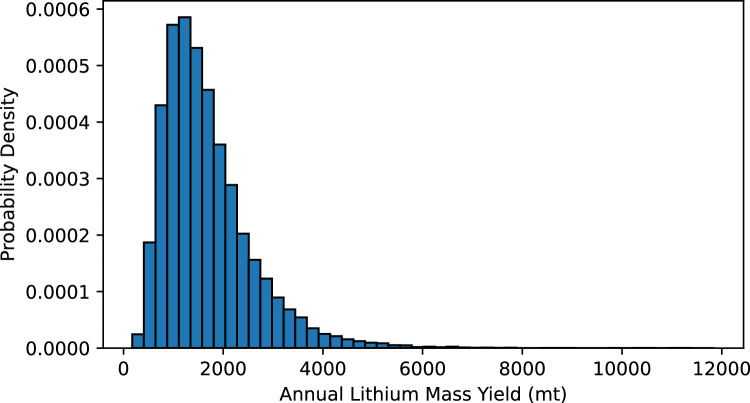
Histogram showing distribution of estimated annual lithium (Li) mass yield from shale gas operations in Pennsylvania. A probability density function (PDF) fit to Monte Carlo simulations (n = 25,000) shows the annual Li mass yield maximum likely estimate is 1160 (95% CI 1140–1180) mt/year.

Herein, we report Monte Carlo estimates of Li mass yields from Pennsylvania’s Marcellus Shale PW. These results will quantify the total annual Li mass yield potential in Pennsylvania from PW, the amount of Li that can be generated from a single Marcellus well in either operating zone (NE PA or SW PA) and patterns of the variables that led to these calculations. The confidence intervals (CI), interquartile range (IQR) and standard deviation (STD) of the associated results are also provided. Additionally, erroneous curve fits were not included in our analysis and may influence real world measurements. Annual, mean PW volume generated in Pennsylvania from 2018 to 2022 was 8.76 × 10^9^ L (STD; ± 5.54 × 10^8^). From this, the maximum likely estimation (MLE) for the total annual Marcellus Li yield is approximately 1160 metric tons (mt) (95% CI 1140–1180) (Fig. 2).

**Figure 3:**
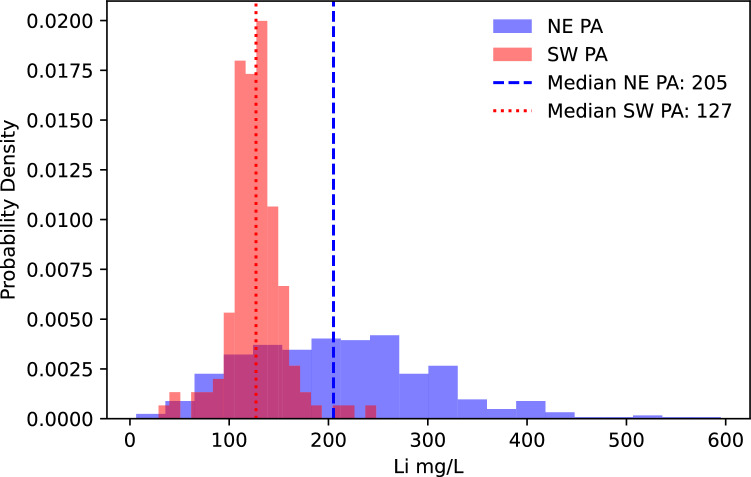
Histogram plot showing regional variance of Li (mg/L) concentrations in Marcellus produced water. NE PA Li distribution is broader and higher (median; 205 mg/L) than the SW PA (median; 127).

**Figure 4:**
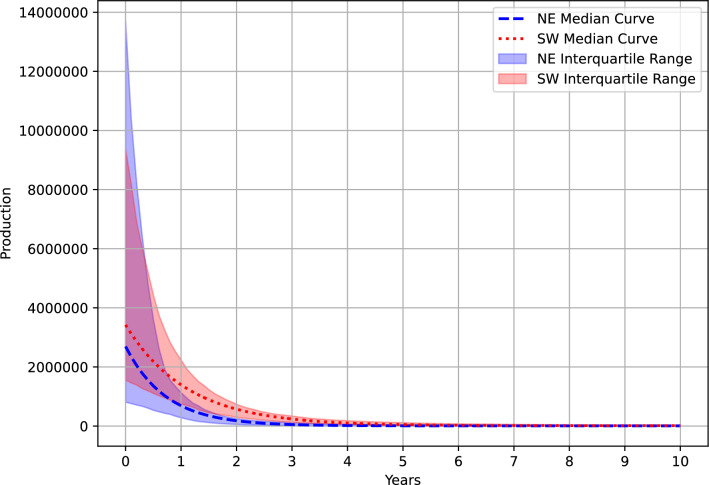
Production decline curve plots for curve fits with an R^2^ ≥ 0.5 for NE and SW PA. Y-axis is in liters and X-axis is time in years from the start of drilling. Median SW PA 10-year cumulative PW production is greater than NE due to a more gradual decline. Wells in the NE producing zone have a higher range of initial water production volumes.

**Figure 5:**
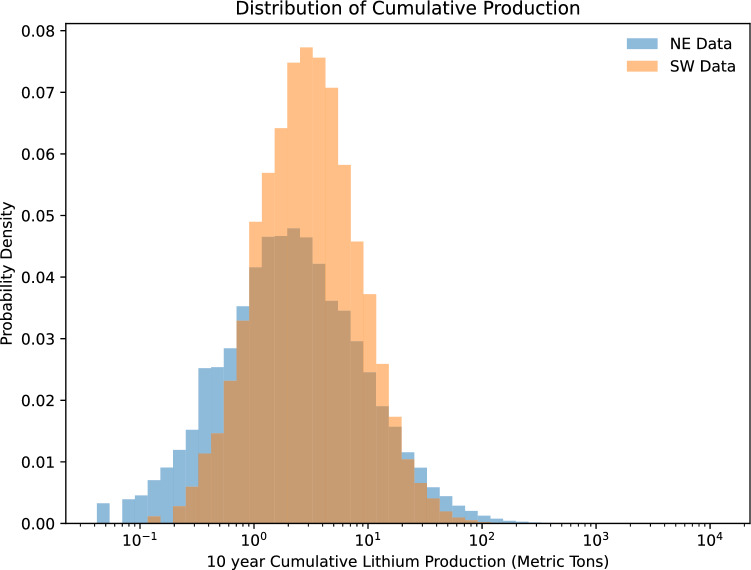
Histogram plot of Monte Carlo simulation results (n = 25,000) of estimated ultimate Li mass yield from a single Marcellus shale gas well over 10-years of assumed continuous production. Regional estimates on lithium yields from a SW PA well is marginally more (~ 33%) than its NE PA counterpart.

Lithium and Mg concentrations and well water production volumes vary between the production regions, which results in marginal differences in MLE Li yields. Produced waters sampled from wells in the NE have a broader distribution of Li concentrations (IQR, 139–267 mg/l; n = 422) with a median of 205 mg/L. In contrast, produced water Li concentrations in SW PA are lower and distributed more narrowly (IQR, 112–140 mg/l; n = 137) with a median concentration of 127 mg/L (Fig. 3). Conversely to Li, more PW is produced in SW PA wells than in NE PA. The median 10-year cumulative PW volume produced by a well in SW PA is over twice that of a NE PA well (4.68 × 10^7^ L and 2.43 × 10^7^, respectively; Fig. 4). Consequently, the 10-year cumulative Li production of a Marcellus well in the NE and SW producing zones (Fig. 5) vary by ~ 33%. The MLE calculations suggest the estimated ultimate lithium recovery (10-year Li mass yield) from individual wells in SW and NE PA are 2.90 (95% CI 2.80–2.99) mt and 1.86 (95% CI 1.86–2.07) mt, respectively.

Additionally, the data reveals significant heterogeneity in magnesium concentrations and Mg/Li mass ratios in PW generated between the two production zones. Median Mg concentrations in the NE PA are roughly half of those measured in the SW PA (NE PA; 1000, SW PA; 2300). Likewise, median Mg/Li ratios vary between the NE and SW PA are 5.4 (IQR, 2.66–7.26; n = 421) and 17.8 (IQR, 14.3–20.7 n = 137), respectively. Descriptive statistics of Li and Mg concentrations, Mg/Li ratios, PW volumes and 10-year Li mass yields are summarized in Table 1.

**Table 1:** Distributions of Lithium (Li), Magnesium (Mg), Mg/Li ratios with simulation results for statewide, northeast (NE PA) and southwest (SW PA) Pennsylvania with 95% confidence intervals (CI).

	*n*	Median	P25	P75	Lithium mass yield	95% CI
Chemical parameters						
NE Mg (mg/L)	421	1000	460	1690	–	
SW Mg (mg/L)	137	2300	1790	2570	–	
NE Mg/Li	422	5.39	2.66	7.26	–	
SW Mg/Li	137	17.8	14.3	20.7	–	
NE Li (mg/Li)	422	205	139	267	–	
SW Li (mg/Li)	137	127	112	140	–	
	–	–	–	–	–	
PW volume and Li mass yield results						
NE 10-year cumulative PW Vol (L)	506	2.43 × 10^7^	–	–	–	
SW 10-year cumulative PW Vol (L)	722	4.68 × 10^7^	–	–	–	
NE PA Li mt/10-year	–	–	–	–	1.96	1.86–2.07
SW PA Li mt/10-year	–	–	–	–	2.90	2.80–2.99
Annual statewide Li mass yield (mt)					1160	1140–1180

Mass is in metric tons (mt).

## Discussion

State-wide MLE of PW resources in the Marcellus suggest this Li source could supply a substantial amount to the domestic markets, though existing PW reuse options need to be considered. Annual domestic Li consumption is estimated at 3,000 metric tons^28^. Astoundingly, statewide Li mass yield estimates suggest Marcellus Shale production wastewater from Pennsylvania could meet 38–40% of current domestic consumption, assuming 100% Li recovery and extraction processes are more cost effective than competing uses for the water. Currently, 95% of the PW generated is reused in ongoing hydraulic fracturing operations and any volumetric offsets from increased treatment would likely be made up with freshwater sources^8,29,30^. Moreover, environmental, social considerations and regulatory structures have spawned investments in water management infrastructure to optimize for PW reuse^29,30^. Typically, PW is transported via a network of pipelines to a central facility where it is minimally treated to remove solids prior to reinjection at other well sites^12,29^. Li extraction from PW would be a more complex process and may increase the environmental footprint of water operations due to added transportation and solid wastes generated from PW treatment. Ultimately, our results show Li mass yields from Marcellus PW are substantial and the added valorization of this waste could offset the needed infrastructure and disposal costs.

Regional variation in PW volumes and chemistry between wells in the NE and SW producing zones likely will impact both the Li extraction method and the ultimate mass of Li generated. Specifically, this study shows that SW PA wells have slower PW decline rates (Fig. 4) and higher ultimate recovery potential (2.90 mt, 95% CI 2.80–2.99; Fig. 5.), compared to a NE PA well (1.96 mt, 95% CI 1.86–2.07). However, SW PA wells only generate, on average, 26–38% more Li when considering differences in PW Li concentrations and the uncertainty of the calculations, despite producing approximately two times the PW volume. Further, extraction of Li from PW with high Mg/Li mass ratios (> 6), such as in SW PA, is less efficient and expensive relative to low Mg/Li extraction methods^31^. The low Mg/Li composition of NE produced waters are comparable to salar brines, such as the Atacama brines of Chile, which are favorable to more economical and sustainable evaporative and distillation Li recovery methods^31,32^. As a result, the higher Li yields from SW PA wells may be more costly to extract due to the lower concentrations and reduced treatment efficiencies due to the high Mg/Li nature of these waters.

Another important consideration in the total Li yield of a reservoir is the well production decline rate. A typical Marcellus well has an 80% decline in production of water within it’s the first 2 years (SI 4.). Sustainable production of Li at volumes reported in this manuscript require continuous addition of new Marcellus wells to supplant older, less productive wells. Advances in artificial lift technologies could improve brine production metrics in older wells and should be a consideration in prolonging the life of this resource. The lift parameter in the model evaluated in this study is a baseline volume of produced water calculated from empirical data and assumed to be resulting from artificial lift installation.

This study estimates that Marcellus Shale related Li yields have potential to make a significant contribution to US domestic consumption with a set of reasonable, conservative assumptions. Even if most likely estimates presented here are off by one or even two standard deviations, the potential production of Li would meet more than 30% of current US domestic consumption. Further, if the estimates are too low, this result becomes an even more promising incentive to properly manage Marcellus PW. The USGS estimate of roughly 96 trillion cubic feet of undiscovered gas in the Marcellus suggests the production lifetime of the formation will exceed several more decades^18^. Future production will likely be on the fringe of the current operational zones, as new territory is developed. North-central PA is underdeveloped and has some of the of the highest Li concentrations included in our analysis (Fig. 1). It seems clear that Marcellus Shale PW has the capacity to provide significant Li yields for the foreseeable future.

## Methods

### Lithium concentration data

Produced waste-water chemical profiles reported to the PA DEP between 2012 and 2023 from unconventional wells targeting the Marcellus Shale were collected^25^. In total, 595 reports were considered from 515 wells. Chemical data were extracted from the PA DEP reports using optical character recognition and custom Python scripts. Two filters were applied to assure data quality: (1) Samples with a major cation/anion charge imbalance >  ± 10% were removed; and (2) only brines (TDS > 35,000 mg/L) were considered to prevent inclusion of dilute flowback waters^33–35^. Lastly, regional PW profiles were sorted and stored based on location using ArcGIS Pro (NE; n = 422, SW; n = 137)^36^. Note that 35 reports were sampled from wells located outside of either producing region and therefore not included in the regional analysis.

### Regional produced water volume calculations

Empirical decline curve analysis (DCA) is a widely used method to forecast the ultimate resource recovery from a hydrocarbon well^37,38^. This study employs DCA methods to forecast and evaluate the regional variability in PW volumes between Marcellus wells in the NE PA and SW PA operating zones, assessed over a decade of presumed continuous production. To do so, we mined Marcellus Shale PW volumes reported to the PA DEP Bureau of Oil and Gas (PA DEP, 2023) by six of the top 10 producers in the Pennsylvania from the years 2009–2022^26^. Top producers were selected based on quantity of natural gas produced, operational footprint (NE PA and SW PA) and continuity of at least one decade of operations. The total well count evaluated from the six operators’ data included in this study account for 42% of wells reporting PW volumes in 2022.

Data processing and regional PW decline rate models for the NE and SW production zones were done in Python 3.9 using the Pandas, SciPy and NumPy packages^39–42^. First, monthly production volume data was parsed and verified to only include wells with the Marcellus Shale designated as the producing formation. Next, production volumes for each well were grouped by their associated API number, the first PW volume was used in the case of a duplicate. Then, well PW production timespans were normalized for each well by calculating the duration of time (months) between well installation (SPUD) and the date the volume was recorded. Non-duplicate, multiple reported volumes sharing a date for a unique API number were summed. The median SPUD normalized Marcellus PW DCA yielded an exponential curve fit that stabilized to a non-zero value approximately six years after the well’s SPUD date (Supplementary Fig. S1). Generally, hydrocarbon well production declines through time, until a point where the bottom hole pressure of the well isn’t sufficient to economically produce hydrocarbons. At this point, operators install an artificial lift mechanism to lift the fluids (hydrocarbon and water) out of the well. A lift factor was included in the decline equation to account for this baseline production These calculations and variable descriptors are detailed in the SI.

Initially, 4798 wells reporting PW waste were evaluated in this DCA. However, a significant number of these wells had insufficient production volume data or reported volumes too noisy to generate accurate curve fits. In extreme cases, the model failed to converge on a fit. A series of quality control measures were applied to improve the success of the curve fits. First, curve fits were only carried out on wells having more than one reported volume and at least one measurement within the first two years from the SPUD date. Second, because Marcellus PW volume decline rates stabilize approximately 6 years from the SPUD of the well, only wells with reported volumes past 6 years from SPUD were considered (n = 2561).

Additional expulsion criteria were used to eliminate curve-fit parameter outliers generated from the DCA. These outlier fits generally arise from data gaps or inconsistencies in the production process rather than variability in the production. Including these fits in the Monte Carlo process artificially inflates the uncertainly. To minimize this inflation, we further filtered the data as follows: First, a goodness-of-fit filter was used to select curve fits with an r-squared (r^2^) of 0.5 or greater. In general, curve fits falling below the 0.5 r^2^ threshold were either positive, flat, vertical or otherwise not decreasing exponentially. Second, inter-quartile range (IQR) threshold analysis was used to identify and remove curve fits that over-estimated the initial production values (Qi)^27^. Outliers exceeding 1.5 of the IQR were removed. While wells with negative calculated lift factor (L) values were not removed, the negative values were converted to zero, as the negatives were considered a relic of the fit rather than actual negative production. After poor fit records were removed, wells with curve fit parameters that passed quality criteria were partitioned into region specific datasets (NE and SW PA) using ArcGIS Pro^36^. In total, 1,228 well decline curves met the quality criteria and fit parameters used in Monte Carlo simulations of production scenarios. Of these, 506 were in the NE and 722 in the SW producing zones of the Marcellus.

### Monte Carlo framework

**Figure 6:**
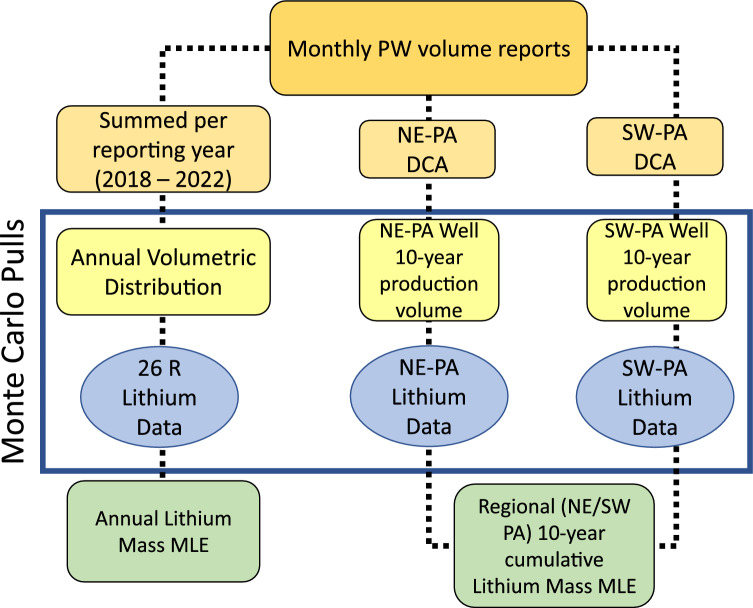
Data process and workflow used in this study.

Monte Carlo (MC) simulations were used to both propagate and mitigate the uncertainty associated with using unrefined datasets to model Li mass yields on statewide and well-by-well scales. All variable “pulls” used in MC simulations were created using NumPy Random Number Generator (RNG) in the Spyder integrated developer environment using Python 3.9 programming language. All distributions generated and employed in our MC simulations were validated using descriptive statistics to ensure a match to the original dataset. A diagram of the data workflow is provided in Fig. 6. Table 2 contains the original data sources and descriptions, distribution type, and RNG parameters (shape and scale) used in this study.

**Table 2:** Distributions of original data, data sources and decline curve fit parameters.

	Variate descriptor	n =	Distribution	distribution type	Numpy Parameters (scale, shape)	Source	
Statewide annual mass yield parameters							
	Lithium concentration	593	Median: 174 (IQR, 125–247) mg/L	lognormal	(5.14, 0.51)	Chemical analysis 26 residual waste chemical analysis reports^25^	
	Annual production volumes	5	8.76 × 10^9^ (STD = ± 5.53 × 10^8^) L	normal		Pennsylvania oil and gas well waste report portal^26^	
Regional 10-year mass yield parameters							
NE PA	NE PA lithium concentration	422	Median: 205 (IQR, 139–267) mg/L	lognormal	(5.3, 0.53)	Chemical analysis 26 residual waste chemical analysis reports^25^	
	Qi (initial production rate)	506	Median = 2.69 × 10^6^ (IQR, 8.11 × 10^5^–1.37 × 10^7^)	lognormal	(16, 1.26)	Curve fit parameter modeled from residual waste volumes^26^	
	D (production decline rate)	506	Median = 0.11 (IQR, 0.054–0.22)	lognormal	(-1.9, 0.65)	Curve fit parameter modeled from residual waste volumes^26^	
	L (lift factor)	506	Median = 6.34 × 10^3^ (IQR, 0–1.23 × 10^4^) L	lognormal	(8.9, 0.80)	Curve fit parameter modeled from residual waste volumes^26^	
SW PA	SW PA lithium concentration	135	Median: 127 (IQR, 112–140) mg/L	lognormal	(4.8, 0.26)	Industry Collaborator provided residual waste chemical analysis	
	Qi (initial production rate)	722	Median = 3.41 × 10^6^ (IQR, 1.55 × 10^6^–9.37 × 10^6^)	lognormal	(16, 0.92)	Curve fit parameter modeled from residual waste volumes^26^	
	D (production decline rate)	722	Median: 0.075 (IQR, 0.048–0.12)	lognormal	(-2.4, 0.51)	Curve fit parameter modeled from residual waste volumes^26^	
	L (lift factor)	722	Median = 1.21 × 10^4^ (IQR, 0–2.98 × 10^4^) L	lognormal	(9.6, 0.94)	Curve fit parameter modeled from residual waste volumes^26^	

Scale and shape values used in NumPy are the mean and standard deviations of the log-transformed dataset.

Annual-statewide estimates of Li mass yields were evaluated using the most recent five years (2018—2022) of total annual Marcellus PW production data. Here, monthly reported volumes were summed for each of the calendar years. NumPy (RNG) was used to fit normal probability distribution functions (PDF) and generate random sample variates of the calculated annual PW volume and the Li concentration distributions described in Table 2. These Monte Carlo samples of volume and chemistry (n = 25,000) were multiplied to derive the most likely estimate of Li mass yields per year from Marcellus operations in Pennsylvania.

A Monte Carlo framework was also used to predict the cumulative PW production and associated Li mass yield for an individual Marcellus well in either the NE PA or SW PA operating zones over a ten-year period. To do this, decline-curve fits and Li concentration data were partitioned into NE PA and SW PA datasets based on the location of origin and used to create separate random sample variates for their respective regions. Given the lognormal distributions of the DCA fit parameters and Li concentrations, shape and scale parameters used to calculate a random distribution for each parameter were taken from the natural log transform of the distribution.

Monte Carlo pulls (25,000) from these RNG generated fit parameter distributions were used to simulate a population of decline curves. Each decline curve was integrated over a 10-year timespan, providing a population of cumulative PW volumes for an individual well. This population of PW volumes were multiplied by a MC pull from a region-specific Li distribution to generate a population of Li mass yields from both NE and SW PA wells. Lastly a probability distribution function (PDF) was fit to the aggregated 10-year cumulative Li mass yields from these simulations and the value with the highest probability density was stored.

Complete data processing, sampling, and modeling descriptions are included in the supplementary information S1.

### Supplementary Information

Supplementary Information.

## Data Availability

The datasets generated and/or analyzed in this study are available on the National Energy Technology Laboratory’s Energy Data eXchange (EDX), https://edx.netl.doe.gov/dataset/lithium-geochemistry-and-regional-production-decline-curves-of-marcellus-shale-produced-water.
