# Supplementary material for: Estimates of lithium mass yields from produced water sourced from the Devonian-aged Marcellus Shale
Source: Sci Rep. 2024 Apr 16;14:8813. doi: 10.1038/s41598-024-58887-x (PMC11021401; doi:10.1038/s41598-024-58887-x)
Supplement: Supplementary file 1 — Supplementary Information. [file 41598_2024_58887_MOESM1_ESM.pdf]

Estimates of lithium mass yields from produced water sourced from the Devonian-aged Marcellus Shale.

Justin Mackey[1,2,3*], Daniel J. Bain[3], Greg Lackey[1], James Gardiner[1], Djuna Gulliver[1], Barbara Kutchko[1]

[1]National Energy Technology Laboratory, Pittsburgh, PA 15236, USA

[2]NETL Support Contractor, Pittsburgh, PA 15236, USA

[3]University of Pittsburgh, Pittsburgh, PA 15260, USA

[*]Corresponding Author. Email Address: Justin.Mackey@netl.doe.gov

Supplemental Information

Data Cleaning

Brine chemistry data were mined from *Form 26R Chemical Analysis of Residual Waste* waste generator compliance reports submitted to the Pennsylvania Department of Environmental Protection (PA DEP) (25 Pa. Code, 287.54). These reports provide chemical analysis of wastes generated on oil and gas wells and well pads in the state of Pennsylvania. Residual Waste Code 802 refers to these as produced fluids, defined as formation fluids recovered from the wellbore, including flow-back and brine.

Chemical analyses of production water (PW) included in *Form 26R Chemical Analysis of Residual Waste* forms were conducted by certified laboratories and governed by the EPA's Test Methods for Evaluating Solid Waste (SW-846) and the Handbook for Analytical Quality Control in Water and Wastewater Laboratories (EPA 600/4-79-019).

Geochemical and production volume data was assessed. Quality checks include verifying that the production volume and/or lithium sample is from a well or pad listing the Marcellus as a

producing interval and cross checking the digitized data with the value indicated on the portable document format (PDF) it was scraped from in northeast Pennsylvania (NE PA). SW PA lithium data was extracted from .csv files provided by an industry collaborator with an operating footprint in southwest Pennsylvania. All sample locations were verified as producing from the Marcellus shale and mapped in ArcGIS Pro. Only samples falling within the SW PA operating zone boundary were selected and used in our SW PA calculations.

A data quality filter was applied to each sample's chemical data (i.e. water sample). Data quality filters were established to prevent the inclusion of dilute flowback waters and non Na-Ca-Cl type brines that were mischaracterized as being from the Marcellus or that have been significantly altered from precipitation reactions in the dataset. Samples meeting any of the following criteria for removal (Supplementary Figure S1) were excluded from the analysis; 1) duplicate values of an existing data point, 2) major element charge balance errors of ± 10%, 3) Ca concentrations less than Mg, 4) major cation concentration ≤ 0 mg/L (i.e., no cation data), 5) total dissolved solids concentration < 35,000 mg/L.. Note the charge balance errors were calculated on major constituents (Na, Ca, Mg, Fe (Tot), Ba, Sr, Cl and Br) using the following equation:

$$CE = 100 * (\frac{CAT - |AN|}{CAT \mp |AN|})$$

Where:

CAT = molar sum of positive charges for major cations

AN = molar sum of negative charges for major anions

Marcellus produced water is typically low in sulfate and not included in the charge balance calculations[1]. SW PA lithium data was extracted from .csv files provided by an industry collaborator with an operating footprint in southwest Pennsylvania. All sample locations were verified as producing from the Marcellus shale and mapped in ArcGIS Pro. Only samples falling within the SW PA operating zone boundary were selected and used in our SW PA calculations.

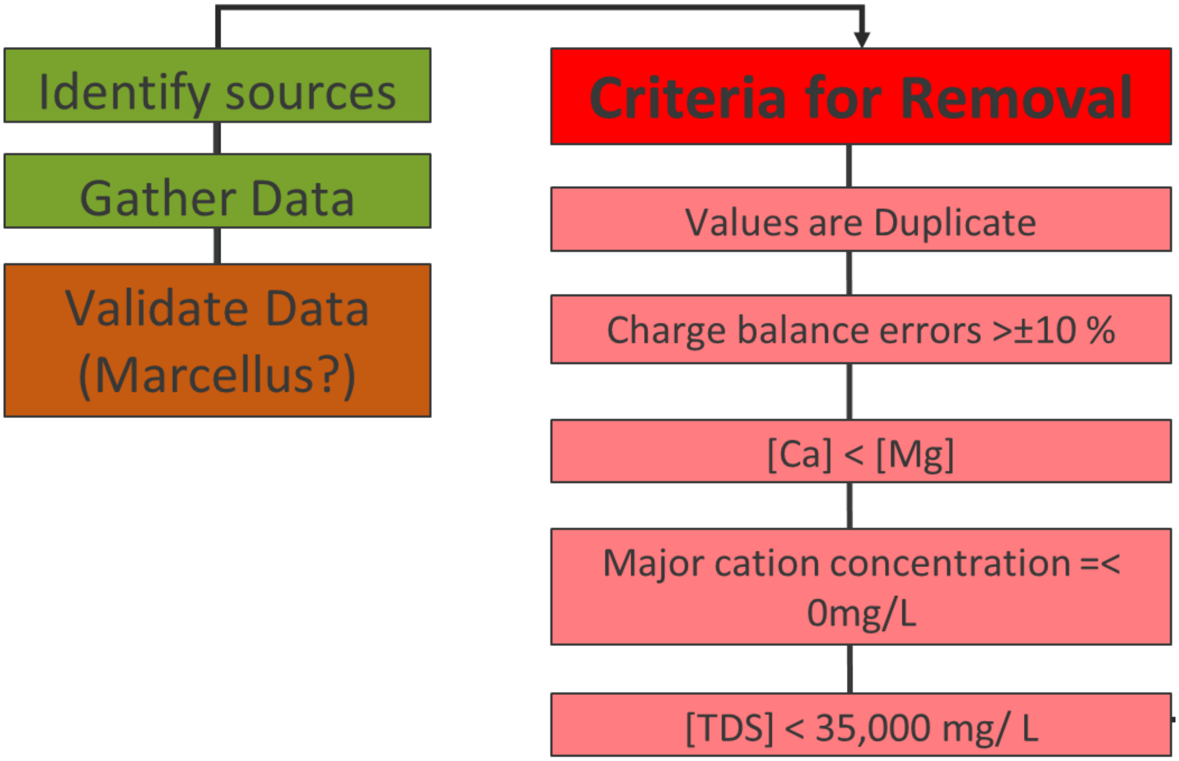

Supplementary Figure S1. Flow diagram depicting criteria for removal applied to chemical profiles of Marcellus Shale produced water.

Distributions of Well Water Production and Lithium concentrations

Decline curve analysis (DCA) was used to estimate the volume of water a Marcellus well generates over the course of a 10-year lifespan. The DCA was conducted on PW data aggregated from waste volume reports submitted by six of the top 10 (by production) operators in the state. These operators were chosen based on quantity of gas production, operational footprint in either the NE or SW PA zones, and long-term (>10 years) continuity of operations within Pennsylvania. The PW dataset is composed of PW volume reports submitted to the PA DEP on a monthly basis as part of a waste generator compliance reporting requirement. Broadly, the raw data access from the PA DEP consists of site information (name, location metrics, etc.), the residual waste type (RWC 802: Produced Water), waste quantity and the date it was reported. We calculated the duration of time between the start of drilling (SPUD) and the date a quantity was reported to the PADEP for each well. Next, quantities were converted to liters and plotted versus time passed since SPUD in months.

The mean quantity of PW at each monthly interval was calculated and plotted using the Seaborn package in Python 3.9 (Supplementary Figure S2). The SPUD normalized, mean PW volume data often yields an exponential decline that tapers to a non-zero value after approximately 6 years (72 months) from SPUD. This mechanism behind the stabilization to a non-zero value is assumed to be the result of artificial lift installation[2]. Therefore, individual curve fits for each well were assessed using an exponential decline curve with the addition of a lift coefficient.

$$\int_0^{10} (Qi * e^{-Dt} + L)\, dt \quad (1)$$

Where:

Q_i is the initial production rate (L/month)

D is the rate of decline (L/month)

t is the time (month’s after the well SPUD date 0

L is the lift factor (L).

Parameters used in the successful curve fits were stored in a data frame for further QC and analysis. Two QC thresholds were applied to the parameter list. First, the R^2 of each curve fit was calculated and only fits with a $R^2 \geq 0.5$ were included to ensure at least 50% of variability in the empirical data was represented by the model. Histograms of the parameters revealed extreme Qi values (10^{23}) in excess of what was observed in the actual dataset. This was likely the result of overestimation the model fit. Therefore, interquartile rank threshold analysis was used to remove fits with over-estimated Qi values that are greater than the IQR threshold of 1.5. The final number of fits for each region are NE PA: 506 and SW PA: 722 (Supplementary Figure S3).

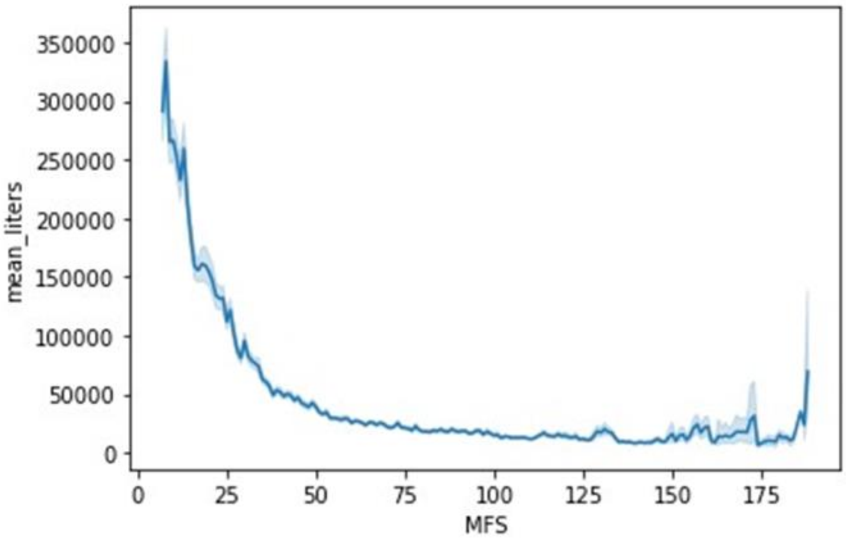

Supplementary Figure S2. Plot of the mean monthly produced water volume generated from Marcellus wells versus time. Data was normalized to the duration of time (in months) from the start of drilling (SPUD) to the date the volume was reported (months from start, MFS).

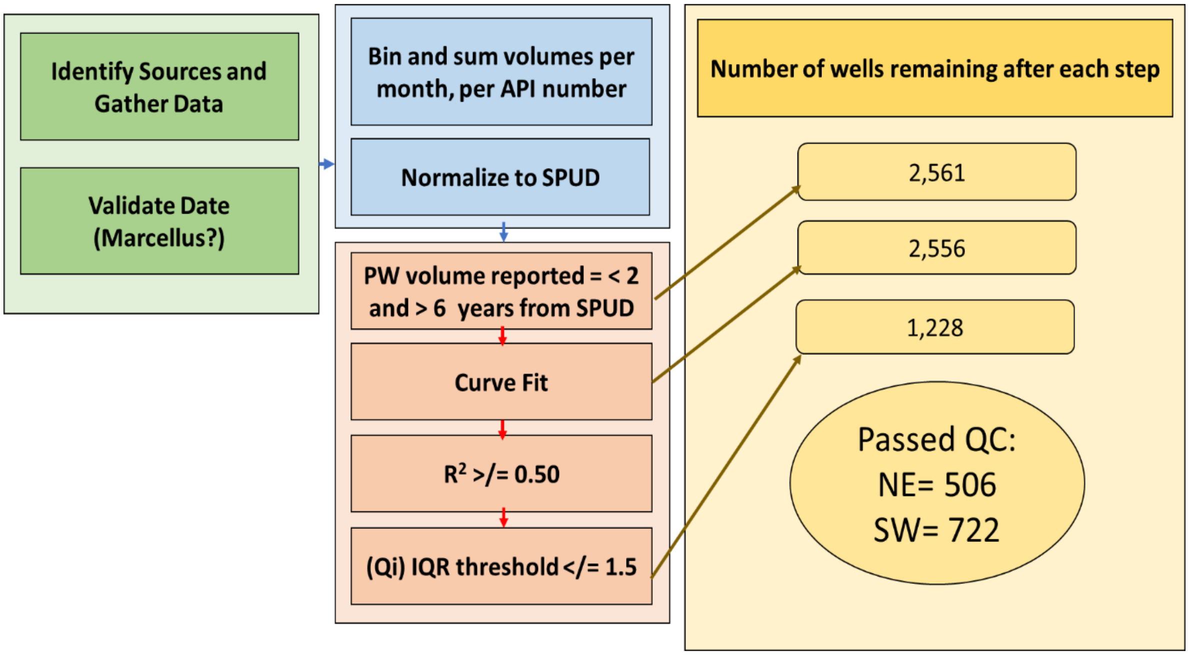

SI Figure 3. Schematic of produced water (PW) volume data processing and QC methodology for decline curve analysis (DCA) on well production data from six of the top 10 gas producers (by volume) in Pennsylvania. 2,561 Marcellus initially considered for DCA. 2,556 of these wells had successful curve fits. Final fit totals with an R^2 </= 0.5 after upper IQR threshold analysis were 506 for NE and 722 for SW Pennsylvania.

Data transformations were used for all lithium concentrations and DCA fit parameters. Measure of skewness analysis shows lithium concentrations and the populations of fit parameters (Qi, D and L) resulting from DCA fits were all lognormally distributed (skewness >0.5). To complete Monte Carlo sampling, the shape (mean) and scale (standard deviation) of the log normally transformed distributions of these datasets were used in NumPy's Random Number Generator (RNG) to generate distributions of parameter sets and lithium concentrations for 25,000 simulations. There were negative lift values created by the DCA fits. These were assumed to be 0 production. Given the structure of the MC framework where a positive lift value is specified, this population of no lift conditions was simulated as follows: During the MC process, lifts were randomly set equal to zero at a rate consistent with regional observations of negative lift values (30% in the SW and 28% in the NE). The final Li data and DCA fit parameter distributions were compared using descriptive statistics and histograms to verify they represent the original data they were generated from (e.g. median Li is within ~10% of original data, and histogram is of similar shape and extent). Original data distributions, NumPy parameters (shape and scale) and data sources are included in Table 1 of the manuscript.

The total annual volume of produced water generated from Marcellus wells in Pennsylvania was calculated for waste compliance reports from the most recent five-year time span (2018 – 2022). (Given continued growth in production, this period is more representative of current conditions than a longer data period). Water volume reports were accessed and downloaded from the Pennsylvania Department of Environmental Protections Waste Generator Portal (PA DEP, 2022). Total Marcellus PW volumes reported to the state were summed per calendar year. The mean and standard deviation of the normal distribution of annual PW volumes were used for MC pulls of annual PW volumes using NumPy RNG.

Produced Water Volume Model Development:

Broadly, annual MLE lithium yields were evaluated by multiplying an MC draw of lithium concentration with the ultimate volume (liters) of PW produced by each well.

Estimates of the lithium mass recovery from a per-well basis were evaluated by simulating PW production over an array of probable production scenarios. Each iteration resulted in an individual PW decline scenario evaluated over a 10 year duration (t). Integration of each curve yields a total cumulative volume of PW generated in the 10 years of simulated production in liters. Previous work suggests lithium concentrations generally stabilize within the first month of production[3]. Therefore, MC simulations assumed an individual MC generated lithium concentration for each 10 year production estimate. The result is a potential lithium mass yield for each decline curve simulation.

Probability distribution functions were fit to the Li mass yield MC results for the NE and SW PA zones and the most probable outcome was inferred from the center of the highest bin in the respective histogram.

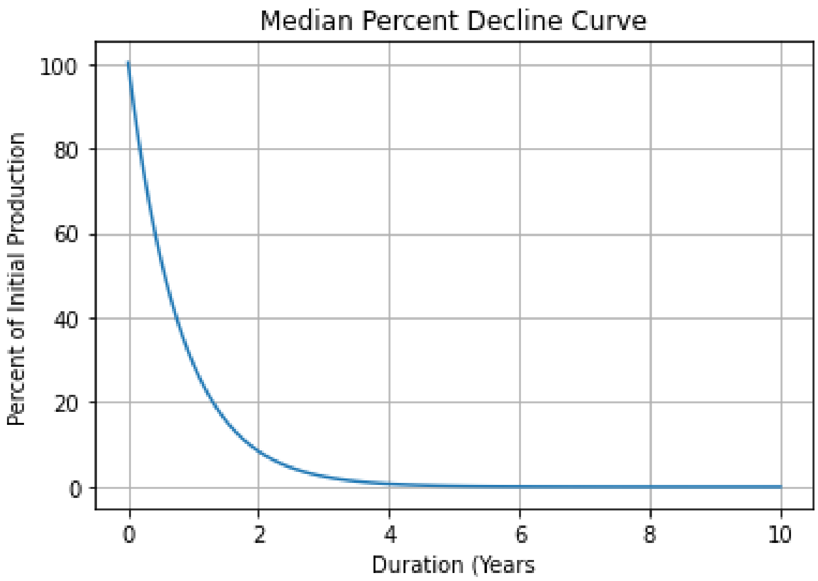

Supplementary Figure S4. Decline curve showing percent of initial production. Curve fit was generated using the median of calculated fit parameters of all wells.

References:

1. Blauch, M. E., Myers, R. R., Moore, T. R., Lipinski, B. A. & Houston, N. A. Marcellus Shale Post-Frac Flowback Waters – Where is All the Salt Coming From and What are the Implications? *SPE East. Reg. Meet.* 221–240 (2009) doi:10.2118/125740-MS.

2. Kolawole, O., Gamadi, T. D. & Bullard, D. Artificial Lift System Applications in Tight Formations: The State of Knowledge. *SPE Prod. Oper.* **35**, 422–434 (2020).

3. Phan, T. T. *et al.* Factors controlling Li concentration and isotopic composition in formation waters and host rocks of Marcellus Shale, Appalachian Basin. *Chem. Geol.* **420**, 162–179 (2016).
